# Crisis and policy imaginaries: higher education reform during a pandemic

**DOI:** 10.1007/s10734-022-00899-5

**Published:** 2022-08-09

**Authors:** Tebeje Molla, Denise Cuthbert

**Affiliations:** 1 School of Education, Deakin University, Melbourne, Australia; 2 School of Graduate Research, RMIT University, Melbourne, Australia

**Keywords:** Crisis discourse, Higher education reform, Job-ready graduates, Policy imaginaries, Australia

## Abstract

Crisis makes bold policy actions possible. In responding to socioeconomic and technological ruptures, policymakers create new imaginaries or revitalise existing ones. With the Australian Government’s Job-Ready Graduates (JRG) reform during the COVID-19 pandemic as an empirical case, this paper shows how crisis instrumentalism and policy imaginaries intersect to effect swift policy changes. Drawing on a thematic analysis of key documents that constitute the JRG reform, we highlight three findings. First, the reformers used a new crisis context to repackage pre-existing policy agendas. Second, in justifying the timeliness of the reform, rather than constructing new imaginaries, the Government reactivated old neoliberal visions of society and the economy. Finally, the reform agendas are characterised by reductionist accounts of the value of university education, a nativist view of the future workforce, and the omissions of key issues: research training, social justice, and the urgency of decarbonising the economy. We close the paper by arguing that *crisis* makes swift *reform* possible to the extent that key actors can mobilise new or pre-existing policy *imaginaries*.

## Introduction

Taking a recent reform in Australian higher education (HE) as an empirical case, this paper explores the interplay between crisis and policy imaginaries. Australia is a federation. While other sectors of the education system fall under State and Territory jurisdictions, the funding of HE is the Commonwealth government’s responsibility. The Federal Government uses this leverage to drive various sector reforms. In August 2014, the Australian Government proposed a major reform (*Higher Education and Research Reform Amendment Bill 2014*) (AHP, 2014) that sought to ‘change higher education in Australia for the better’. The Bill aimed at deregulating the HE sector to meet the skill needs of the economy. In introducing the Bill to the Parliament, the Minister of Education declared: ‘Deregulation is the only way to respond to what students and employers want’ (Pyne, 2014, para.13). The university sector expressed strong opposition to the measures of fee deregulation (Borrello & Giul, 2014), and the Bill failed to pass the parliamentary debate. In December 2014, Education Minister Christopher Pyne reintroduced an amended bill (*Higher Education and Research Reform Bill 2014*) (APH, 2014). The revised proposal was not significantly different from the original Bill. The focus remained on shifting the financial burden to students. The amended Bill again triggered sector-wide opposition and floundered in the Parliament. In May 2017, Education Minister Simon Birmingham announced the *Higher Education Support Legislation Bill 2017* to restructure the higher education funding program (APH, 2017). Echoing the long-held market logic that justifies increased user payments on the assumption of high private benefits of university education (Marginson, 1997), the Bill sought to introduce a new funding arrangement that increases student contribution amounts in selected fields. The proposal faced strong opposition from the sector (Savage et al., 2019). For the third time, the reform failed.

The COVID-19 pandemic presented the Government with an opportunity to make its fourth attempt at reforming HE funding. The pandemic exposed the vulnerability of Australia’s HE sector (Blackmore, 2020). As Australian universities had come to rely heavily on fees from international students for research funding (as education is one of the nation’s biggest exports (Welch, 2022)), the pandemic caused significant disruptions in the sector. With the closing of international borders, the sector lost billions of dollars in income, and universities shed about 35,000 jobs (Littleton & Stanford, 2021), with potentially thousands more losses to come. The intervention of the Government was more important than ever. In other words, the COVID-19 crisis created greater public openness to change, and the Government took advantage of the moment to introduce bold political measures. Essentially, the crisis created a ‘policy window’ (Kingdon, 2013). The political dictum ‘don’t waste a crisis’ was effectively enacted.

In August 2020, Education Minister Dan Tehan introduced the *Higher Education Support Amendment (Job-Ready Graduates and Supporting Regional and Remote Students) Bill 2020*. This Job-Ready Graduate (JRG) reform package contains a suite of significant changes to address what the Government saw as a ‘misalignment between the cost of teaching a degree and the revenue that a university receives to teach it’. In promoting the reform, the Government evoked a crisis narrative. As shown in the analysis below, the policy selling point was that the health crisis (COVID-19) coupled with fast-paced technological changes in the workplace poses a risk to national economic productivity and competitiveness. The reform was represented as a means to ensure that the HE sector plays a vital role in helping the nation avert the economic crisis. The Bill passed the Parliament within two months, and the Government secured the necessary legislative tool to deregulate university fees.

Crises may prompt shared visions for new or alternative futures or what we call policy imaginaries. This paper pays special attention to how the *crisis discourse* is invoked in the reform process and what *futures are imagined* to be realised through the reform. Imaginaries signify the ability to ‘see in a thing what it is not’ (Castoriadis, 1975/1987, p.127). In problematising the Job-Ready Graduates (JRG) reform, we ask what the policymakers saw in the crisis: what were their imaginaries? In so doing, we explore problem areas, policy responses, and the imagined futures of the reform. The aim is to understand how the Government mobilised a crisis narrative to (re)articulate specific policy imaginaries that inform the reform. The following research questions guide our analysis:

- How are the problem areas that necessitate the JRG framed?
- What policy responses are put in place in response to the crisis areas?
- What are the policy imaginaries that the reform seeks to achieve?

Using the concept of *policy imaginaries* to understand the deployment of *crisis* narratives to drive *reform* is a novel approach to the critical analysis of higher education policy. Why do we need to study policy imaginaries now? Policy imaginaries structure our conception of reality and the collective response to it. Investigating policy imaginaries allows us to appreciate what is detected as a risk and/or what is envisioned as a desirable future for society. For instance, our analysis shows a degree of *crisis instrumentalism* at work in deploying the COVID-19 crisis to effect a long-held policy agenda.

The remainder of the paper is organised into four main sections. In what follows, we discuss the theoretical perspectives that inform our analysis, namely crisis and social imaginaries. The second section covers the methodological approaches of the study. The third section presents a descriptive account of problem areas, policy responses, and imagined futures of the reform. In the fourth section, we problematise the reform, specifically highlighting its reductionist tendencies, nativist orientations, and issue omissions. In closing, the paper summarises key findings and reflects on alternative policy ideas.

## Crisis and imaginaries: an analytical framework

Since the second half of the 2010s, we have witnessed a series of crises that called for public action. These include the rise of extreme nationalism and subsequent political ruptures in traditionally liberal democratic societies (Castells, 2018), climate change and its associated predicaments in politics and epistemology (Latour, 2018), and, more recently, the COVID-19 global pandemic and associated socioeconomic turbulence (Schewab & Malleret, 2020).^1^

A crisis is an expression of disruptive change. Fassin (2021) defines crisis as ‘dramatic ruptures into the normal course of things that […] call for urgent solution’ (p.265). Crisis erodes confidence in the status quo, resulting in the urgency to return to regularity and making drastic reforms possible and difficult decisions acceptable. To use Friedman’s (1962/2002) framing, when a crisis hits a system or society, ‘the politically impossible becomes politically inevitable’ (p. ix). In other words, a crisis enables policymakers to persuade the public that ‘there is no escape from a large and painful dose of medicine’ (Bruno, 1993, p.79). But a societal or systemic rupture is not a crisis until it is framed as such. As Prudham (2005) notes, ‘there is no crisis without someone to call it one [and] how it is named will influence how it is addressed’ (p.21). Relatedly, whether policymakers view the rupture as a ‘public problem’ and how they respond to it depend on their positionalities (i.e. their values and interests).

Crisis triggers change partly through generating or activating imaginaries. In the moment of crisis, as the usual courses of action become irrelevant, new measures and possibilities may become imaginable. When a crisis is well aligned with imaginaries (new or existing), policy change is swift and largely consensual. In this paper, we are particularly interested in understanding how crisis triggers change by generating new policy imaginaries or reactivating existing ones. But what are imaginaries?

Imaginaries are essential elements of our collective life. Appadurai (1996) observes that to extend our horizons of possibility, we engage in ‘the work of the imagination’—we actively construct a ‘landscape of collective aspirations’ that depicts how we see our future and its realisation (p.31). Imaginaries manifest in *a shared framework of tacit belief* within which people make sense of their current practices and future trajectories. For Taylor (2004), imaginaries are ‘the ways people imagine their social existence, how they fit together with others […], and the deeper normative notions and images that underlie these expectations’ (p.23). Imaginaries may be created by people in power and thinkers; they may arise from theoretical or ideological positions and infiltrate into the public narrative and form a shared vision (Jasanoff, 2015; Taylor, 2004). Imaginaries take roots through *narratives* (e.g. causal analysis and moral claims) that anchor shared concerns and *framing devices* (e.g. metaphors and catchphrases) that represent desired futures and orient actions. Bessant (2017) explains how the interplay between crisis and collective imaginaries makes policymaking possible:

Any crisis involves a combination of ‘something’ that is happening (or is believed to be happening) and the socially constructed and shared ‘frames’ available to a community to make sense of what is happening. In this way, policymaking communities change their policy paradigms because they have made new sense of a ‘problem’ and the range of ‘solutions’, typically for various ‘political’ reasons. (p.61)

Ideally, imaginaries enable us to ‘put the actual under the light of the possible’ and in so doing, they ‘do the work of crisis without crisis’ (Unger, 2001, p.iii). However, in reality, imaginaries often take root in the context of crisis. Those who wait in the wings with well-formulated alternatives are more likely to succeed in offering a way out when crises emerge (Friedman, 1962/2002). Policy imaginaries may move from top-down (e.g. in the form of political narratives and theories) as well as from bottom-up (e.g. in the form of social movements that draw on shared conceptions about what society is and should be). In the case of the JRG reform, the process proceeded with no genuine public consultation: the Government allowed just five working days for public submissions; it introduced and successfully legislated the reform within two months.

The concept of the imaginary has been invoked to understand how society ‘imaginatively institutes’ itself through laws and narratives (Castoriadis, 1975/1998), explain the formation of nations as ‘imagined communities’ (Anderson, 1983), analyse cultural and technological forces of globalisation (Appadurai, 1996), trace the rise of individualism and shifts in the moral order in modern Western societies (Taylor, 2004), explicate the emergence of a global political belief system (Steger, 2008), and show the role of elites in technological visions and policies in society (Jasanoff, 2015). Drawing on these leading scholars of social and political imaginaries, we focus on policy imaginaries in the recent reform of Australian higher education. If imaginaries are ‘collectively held, institutionally stabilised, and publicly performed visions of desirable futures’ (Jasanoff, 2015, p.4), one of the key expressions of such visions is policy. Policies exemplify how shared imaginaries can become ‘a staging ground for action’ (Appadurai, 1996). But not all imaginaries are equally desirable. Social imaginaries may reproduce privilege and marginalisation.

Drawing on the literature outlined above, we define *policy imaginaries* as shared visions of desirable futures animated by communal understandings of present challenges or opportunities. While social imaginaries refer to broader collective visions, policy imaginaries are about those shared visions that guide and are realised through planned public actions and initiatives. When education systems are seen as insufficient to fulfil the socioeconomic expectations of society, crisis talk takes hold and forms a policy imaginary. Policymakers reiterate the crisis and the desirable futures expected to be realised through the policy. In this paper, we aim to understand the tacit framework of belief that underlies the JRG reform in Australia and to problematise its performance of crisis instrumentalism.

## Methodology and data

Policymaking entails framing problems and naming instruments; it is an act of representing issues in ways that resonate with the supposed priorities and aspirations of the public. The interplay between crisis and policy imaginaries is particularly evident when governments respond to disruptive events with reforms that outline ‘what needs to change’ and signal ‘what we [imagine] ourselves to be’ (Bacchi, 2009, p.158). It is also imperative to note that imaginaries do not hang in the air. They are attached to symbolic representations (e.g. policy narratives) and material objects and practices (Valaskivi & Sumiala, 2014). The preceding discussion shows that, in transforming individual visions into shared objectives, politicians and policymakers use public reasoning (and symbolic representations such as metaphors, images, catchphrases, and dramatic stories) to disperse their ideas. Through patterned discursive performance, they ritualise certain views and naturalise ways of ‘thinking about possible worlds’ (Jasanoff, 2015, p.23). Understanding policy imaginaries and crisis representation require interpretive methods that attend to the representation of the problem and the alternative futures to be realised through collective action. The role of the policy analyst is to grasp what sorts of changes are envisioned, the underlying assumptions of the proposed changes, and whose voices are excluded from consideration. In introducing the JRG reform, we ask, what is the Government hoping to change—what desirable futures are imagined in response to the ‘crisis’ identified?

For this paper, qualitative data were generated through a review of three policy documents, namely the *Job-Ready Graduates Discussion Paper* (DESE, 2020a), the *Department of Education and Training’s Submission to the Senate* (DESE, 2020b), and the *Higher Education Support Amendment (Job-Ready* 6 *Graduates and Supporting Regional and Remote Students) Act 2020* (PCA, 2020). The document review entailed tracing the content and contours of policy imaginaries in the JRG reform, with particular attention to articulations of anxiety associated with the present and hope for the future. In making sense of the crises and imaginaries articulated in the reform, we applied an inductive thematic analysis approach, which allows deep theoretical sensibility and critical engagement with data (Braun & Clarke, 2021). After data familiarisation, each document was read and colour-coded line by line. We then identified key statements, mapped emerging patterns, and constructed ten themes that capture aspects of crisis discourse and policy imaginaries in the documents. Finally, we regrouped the themes into three categories: crisis areas, policy responses, and imagined futures (see Table 1). Following *descriptive* accounts of these categories, we *critically* engage with the reform and shed light on reductionist accounts of the value of university education, a nativist view of the future workforce, and the omissions of key issues.

**Table 1:** A summary of the inductive analysis used in the study

Segments of key statements extracted from selected policy documents	Themes	Categories
‘Arresting the decline in labour force productivity growth that Australia has experienced in recent years must be a top national priority in the recovery period.’ (DESE, 2020a, p.5) ‘We must do what we can now to ensure there is a pipeline of skilled workers in these priority fields, working to prevent future skills shortages the impact on the Australian economy.’ (DESE, 2020a, p.22) ‘Australia’s economic growth has been affected by the pandemic, and our labour force needs are changing quickly.’ (DESE, 2020b, p.7) ‘With low-skilled jobs increasingly automated, the bar for skills and education requirements to enter many professions and occupations will continue to rise.’ (DESE, 2020b, p.14)	Skills shortage and employability	*Crisis areas*
‘Current funding system doesn’t incentivise study in areas of industry need.’ (DESE, 2020a, p.11) ‘There is no dedicated stream of funding that supports university engagement with businesses and industry.’ (DESE, 2020a, p.12) ‘industry groups are seeking more from graduates as they enter the workforce.’ (DESE, 2020b, p.36	Weak industry linkage	
‘A population bulge colloquially known as the ‘Costello baby boom’ is moving through the population and will impact university enrolments in the mid-2020s.’ (DESE, 2020a, p.13) ‘Our current funding arrangements will come under increasing pressure in the 2020s as a larger cohort of 18 year olds move into higher education.’ (DESE, 2020a, p.11) ‘The gap between funding growth and population growth under current settings will not allow the system to develop capacity in pace with future demand.’ (DESE, 2020b, p.18)	Population growth	
‘The higher education attainment rate in regional Australia is significantly lower than in metropolitan areas.’ (DESE, 2020a, p.14) ‘The Government recognises that more work must be done to close the attainment gap between students from regional Australia and those in metropolitan areas.’ (DESE, 2020b, p.5) ‘Beyond general demand, there is a geographical attainment gap that needs to be addressed.’ (DESE, 2020b, p.11)	Geographic disparities	
‘Within the higher education funding system there are opportunities to […] provide incentives to produce graduates in disciplines that support the national interest.’ (DESE, 2020a, p.7) ‘Post-COVID-19, there will be changes to the labour market. It is clear we will need more job-ready graduates in health care, teaching and STEM related fields, including engineering and IT.’ (DESE, 2020a, p.14) ‘The reforms will incentivise students to make more job-relevant choices, that lead to more job-ready graduates, by reducing the student contribution in areas of expected employment growth and demand.’ (DESE, 2020b, p.20)	Price signalling	*Policy responses*
‘Improving industry-university collaboration in teaching and research is equally critical to ensuring graduates leave the higher education system with the skills and experience needed to succeed in the workforce.’ (DESE, 2020a, p.5) ‘provide a dedicated funding stream for universities to carry out engagement with industry, develop industry-relevant course material, optimise the course mix for local economies, promote the teaching of STEM subjects and provide work-integrated learning opportunities for students.’ (DESE, 2020b, p.28) The purposes of universities include ‘the engagement with industry and the local community to enable graduates to thrive in the workforce.’ (Australian Government, 2003, Division 2[b] [iv])	University-industry Collaboration	
‘The higher education system will need to meet an unexpected spike in demand from school leavers.’ (DESE, 2020a, p.3) ‘This additional funding [for new student places] will ensure the Australian higher education system is better placed to deal with the anticipated increase in demand for higher education.’ (DESE, 2020b, p.51) ‘The focus of the package is to grow the number of university places for domestic students by 100,000 by 2030 and encourage students to consider Job-ready education pathways.’ (DESE, 2020b, p.5)	Increase university places for domestic students	
‘We need a regional, rural and remote education strategy that will lift attainment rates.’ (DESE, 2020a, p.28) ‘The regionally focused measures are targeted to provide opportunities for regional and remote students to attend university, and to support additional investment in regional universities to boost regional development.’ (DESE, 2020b, p.22) ‘Direct growth to the regions, ensuring more regional and remote students have access to a university place.’ (DESE, 2020b, p.33)	Regional funding	
‘A strong economic recovery will depend on knowledge-intensive jobs held by Australians who are highly skilled, creative and flexible.’ (DESE, 2020a, p.3) ‘Tertiary education is crucial to the future of Australia’s regional economies, ensuring our nation has individuals with the necessary skills for the jobs of the future.’ (DESE, 2020a, p.28) Higher education will play an important role in ‘increasing the supply of highly skilled and knowledgeable workers able to drive innovation within business, develop and adapt to new technologies, and undertake basic and applied research.’ (DESE, 2020a, p.5) ‘Improving educational outcomes in regional and remote Australia and boosting the contribution made by regional Australia, including the capacity of education institutions, will help build the nation, increase productivity and promote decentralisation.’ (DESE, 2020b, p.13)	Economically productivity nation	*Imagined Futures*
Mission based compacts between universities and the Government must include ‘a statement of the provider’s strategies for improving equality of opportunity in higher education’ (Australian Government, 2003, Division 19-110 [3] [e]) ‘Provide financial incentives and introduce demand driven funding for regional Indigenous students and provide more places for regional university campuses.’ (DESE, 2020a, p.11) ‘Indigenous students from rural and regional Australia who are admitted to their university of choice will have a guaranteed Bachelor-level CSP at university.’ (DESE, 2020b, p.46)	Socially just higher education	

*DP* Discussion Paper on Higher Education Reform Package 2020; *HSE Act* Higher Education Support Act; *DET Submission* Department of Education and Training Submission to the Senate Standing Committee on Education and Employment

## Problem areas, policy responses, and imagined futures

For Edelman (1977), at the core of policymaking is ‘the linguistic structuring of social problems’ (p.26) and ‘how the problem is named involves alternative scenarios, each with its own facts, value judgments, and emotions’ (p. 29). These values and facts help define problem areas and desirable futures. In a time of crisis, a policy narrative has elements of both *temporality* (triggering a sense of urgency) and *affectivity* (triggering anxiety) (Fassin, 2021). By creating a shared perception of threats or opportunities, the crisis narrative makes drastic policy changes possible.

In this paper, we show that the Australian Government capitalised on the COVID-19 pandemic to invoke its crisis discourse, justifying the urgency of the JRG reform in part by highlighting the economic downturn that resulted from the pandemic (DESE, 2020a, b). At the core of the reform package is a restructuring of the HE funding system, which has been a goal of the Liberal Coalition government since 2014. This long-standing goal is revivified in the context of the COVID crisis with new emphases: a focus on the employability of graduates, incentives for study in select fields through new fee schedules, and a recasting of equity exclusively in terms of opportunities for regional students and universities.

Beyond the profound changes in funding areas, the JRG reform has resulted in significant alterations to the nation’s higher education law (the *Higher Education Support Act 2003*). It has strategically extended the purpose of universities while arguably reducing their role to that of a subset of the economy. Universities are now required to develop strategies for ‘engaging with industry’ and ‘improving equality of opportunity’ (Division 19–110 [3] [d-e]). Our review highlights specifically what was seen as *a problem*, what *policy responses* were introduced, and what was conceived as a *desirable future.* These points are discussed in turn.

### Problem areas

Using the COVID disruption as an opportunity, the reform identifies specific crisis areas associated with the HE sector: skills shortage, weak industry linkage, growth in school leavers, and a geographical attainment gap.

The reform emphasises the *shortage of skill sets* relevant to the ‘digital economy’ and future productivity. The Government sees skills shortage as caused by a funding system that ‘does not incentivise study in areas of industry need’ (DESE, 2020a). The restructuring of the funding system aims to direct students to take courses that lead to ‘jobs of national importance’ (DESE, 2020a). The assumption is that with increased automation, ‘the bar for skills and education requirements to enter many professions and occupations will continue to rise’ (DESE, 2020b, p.14). The Government further stresses, ‘We must do what we can now to ensure there is a pipeline of skilled workers in these priority fields, working to prevent future skills shortages the impact on the Australian economy’ (DESE, 2020a, p.22). The reform calls for increasing job growth in science, technology, engineering, and mathematics (STEM) fields.

Skills shortage is also attributed to what is viewed as ineffective university-industry collaboration. Measured in terms of course alignment, work placement opportunities, and collaboration in research and teaching, *industry linkage* is seen as weak. The reformers argue that students are graduating without the relevant skills and knowledge required in the workplace (DESE, 2020a, b). The *geographical attainment gap* is also identified as a crisis area requiring a policy response. The reform makes additional funding for closing the HE attainment gap between students in metropolitan parts of Australia and those from regional and rural areas (DESE, 2020a). The social justice agenda is narrowed to the economic implications of low HE attainment rates in regional and rural areas.

Another impetus for the reform is the expected spike in university demand from school leavers. This *growth in school leavers* is a consequence of another policy intervention in the early 2000s. Through a baby bonus scheme, treasurer Peter Costello encouraged Australians to have three children: ‘one for mum, one for dad, and one for the country’.^2^ What followed was the so-called the Costello baby boom generation that attained university age in the 2020s. The JRG reform acknowledges: ‘Our current funding arrangements will come under increasing pressure in the 2020s as a larger cohort of 18-year olds move into higher education’ (DESE, 2020b, p.11). For the Government, without policy measures to allocate funding for additional university places, the ‘baby bonus’ generation might struggle to access HE (DESE, 2020b).

### Policy responses

A crisis represents ’a moment of decisive intervention’ (Hay, 1996, p.253) and makes swift policy action possible. Friedman (1962/2002) writes: ‘Only a crisis – actual or perceived – produces real change. When that crisis occurs, the actions that are taken depend on the ideas that are lying around’ (p.xiv). Friedman’s second sentence is insightful here. When crisis strikes, actors who have ideas in hand are more likely to succeed in making sweeping legislative changes mainly because (a) shock makes it easy for them ‘to sell the need for intervention to the public’ (Klein, 2008, p.310) and (b) things are difficult to fully grasp once they reach ‘crisis proportions’ (Morin, 1999). A politically mediated perception of public issues achieves primacy during a crisis, opening opportunities for reimagining the future. Without a crisis that threatens the status quo, Kuipers (2006) suggests, policy actors may struggle ‘to break the conservative influence of established institutions and their advocates’ (p.180).

Policy may be seen as a constitutive element of, and an instrument for, translating broad social imaginaries. What makes a policy imaginary distinctive from other forms of social imaginaries is that often the former responds to crisis and outlines strategies for achieving desired futures. The policy imaginaries encapsulated in the JRG reform are expressed in the Government’s effort to ensure that the HE sector is ‘fit for the future’ in terms of supporting economic productivity and social inclusion. The Government uses price signalling as a policy instrument to orient universities and students toward qualifications relevant to ‘jobs of the future’. The notion of price signalling entails the reduction of the student contribution in areas of identified future employment growth and demand. For example, students will pay less to study teaching, nursing, clinical psychology, English and languages, agriculture, maths, science, health, architecture, environmental science, IT, and engineering (DESE, 202b). The Government maintains:

The reforms will incentivise students to make *more job-relevant choices* that lead to *more job-ready graduates*, by reducing the student contribution in areas of expected employment growth and demand. This will also help student to receive an education that sets them up *for future success* – because if graduates succeed, they will support an *economic recovery* that benefits all Australians. (DESE, 2020b, p.20, emphasis added)

Ensuring strong *university-industry collaboration* has been a policy agenda for many years (Cuthbert & Molla, 2015a, b; Molla & Cuthbert, 2019). However, as there was no dedicated funding stream for this purpose, universities often had to direct resources from student funding to undertake collaboration activities. To address this funding gap, the reform stipulates the establishment of a dedicated *National Priorities Industry Linkage Fund* (DESE, 2020a, b). The partnership between universities and local industries creates work-integrated learning opportunities for students, internships, placements, the development of industry-relevant course material, and close collaboration in course development and research activities (DESE, 2020a). The Government stresses:

Increased collaboration offers benefits for everyone, allowing students to gain the skills they need for the workforce, providers to respond to the demand for these skills from students and employers, and employers to benefit from job-ready graduates and the new, ground-breaking research and development needed to drive productivity growth. (DESE, 2020b, p.5)

Maintaining a balance between population growth and university funding is seen as a critical policy goal. The Government argues that without reform, ‘there will not be enough university places for prospective Australian students’ (DESE, 2020b, p.18). To allay this risk, the reform seeks to increase university places by 100,000 by 2030 (DESE, 2020b). Hence, to meet the spike in demand from school leavers in the 2020s, universities will need additional funding. Finally, as noted above, the JRG reform sees geographical disparities in HE attainment as a critical issue. In response, the reform introduces measures to enhance the HE participation of regional and rural students. The reform provides *extra funding* to encourage students from regional and remote areas to undertake university studies. The support includes scholarships to cover relocation costs and additional funding ($400 million) for regional universities to widen alternative pathways for this group of students. The reform aims at lifting the tertiary education attainment of people from regional, rural, and remote communities (DESE, 2020b).

### Imagined futures

Through collective imaginaries encapsulated in stories and myths, society projects desirable goals to achieve imagined futures. As Harvey (2000) notes, clarity of imaginaries enables us ‘to act as *conscious architects of our fates* rather than as “helpless puppets” of the institutional and imaginative worlds we inhabit’ (p.156, emphasis added). Jasanoff and Kim (2009) speak of social imaginaries as residing ‘in the reservoir of norms and discourses, metaphors and cultural meanings out of which actors build their policy preferences’ (p.123). In our analysis of the JRG policy text corpus, we pay special attention to which issues are framed as crisis areas and for which desirable futures are envisioned, including collective beliefs about how HE functions and to what ends. In this regard, the reform aims to achieve productivity and narrowly conceived equity goals—to realise a future comprising an *economically productive nation*, a *socially equitable higher education*, *and highly skilled Australians in Australian jobs.*

The economic imperative of the reform is stark. The JRG reform aims to ensure that Australia remains *economically productive and competitive*. The HE sector is seen as a subset of the economic sector—it is expected to increase ‘the supply of highly skilled and knowledgeable workers able to drive innovation within business, develop and adapt to new technologies, and undertake basic and applied research’ (DESE, 2020a, p.5). The reform seeks to support economic competitiveness by encouraging universities to produce graduates for jobs of national importance such as teaching, nursing, and the STEM fields. Job-ready graduates are those who not only meet the work requirements of today but also the needs of the future workforce (DESE, 2020a). The restructuring of the higher education funding system aims at, among other things, providing ‘incentives to produce graduates in disciplines that support the national interest’ (DESE, 2020a, p.7) and thereby ensuring that ‘all Australians have the job-ready skills in a challenging labour market’ (DESE, 2020b, p.17). In Australia, governments of all political persuasions, at least rhetorically, acknowledge the role of higher education in the nation’s progress. Even so, the current instrumentalist and narrow account of the role of HE represents a significant departure from the legacy of the Liberal Prime Minister and architect of the post-World War II expansion of Australia’s HE system, Robert Menzies. In 1957, in introducing the Murray Report to the Parliament, then Prime Minister Menzies noted:

The social, scientific, economic and industrial complexities of Australia today are largely beyond the imagination of forty years ago. Great skill achieved after high training is no longer to be regarded as something to be admired in a few. We must, on a broad basis, become a more and more educated democracy if we are to raise our spiritual, intellectual, and material living standards. (Menzies, 1970, p.86)

Likewise, social justice goals have long been part of Australian HE, albeit in varying degrees (Molla, 2021). Focusing on regional students and universities, the JRG reform reiterates a commitment to equity in its imaginary of Australia’s future HE sector. Primarily, but selectively, responding to the problem of inequality in HE attainment highlighted in subsequent commissioned reports (e.g. the Performance-based Funding report [2019]; the Productivity Commission report [2019]; the National Reginal, Rural and Remote Tertiary Education Strategy [2019]), the JRG reform requires equity measures mainly focused on closing the educational attainment gap between regional and metropolitan areas. The reform provides financial incentives and introduces ‘demand-driven funding for regional Indigenous students and provide more places for *regional university* campuses’ (DESE, 2020a, p.11). Furthermore, the mission-based compacts between universities and the Government include ‘a statement of the provider’s strategies for *improving equality of opportunity* in higher education’ (Australian Government, 2003, Division 19–110 [3] [e], emphasis added). The compacts are seen as instrumental for directing universities toward specified national agendas. In equity areas, as stipulated under Sect. 30(2) of the *Higher Education Support Act 2003* (amended in January 2021), examples of achieving national priorities entail ‘increasing the number of *particular kinds of persons* undertaking courses of study’ and ‘increasing the number of *persons in particular regions* undertaking courses of study’ (Australian Government, 2003, emphasis added).

The reform is guided by the expectation that *universities should support the nation’s economic productivity* and that *public spending on education should aim at maximising economic returns*. These are recycled imaginaries. It is evident that COVID was both a crisis seized opportunistically and an opportunity lost by the Australian Government for visionary reform. As outlined in the "Introduction" section of this paper, the policy options were essentially set years before the COVID-19 crisis, and the desirable futures outlined in the reform are largely a rehearsal of old political arguments for increased efficiency, productivity, and accountability.

## Problematising the reform

The JRG reform is characterised by issue reduction and omission, recycled imaginaries, and nativism. The *reductionist aspect* of the reform is evinced in the emphasis placed on the economic returns of the HE sector. The JRG reform assumes a future with a settled political and social sphere, where these are imagined as existing beyond the economic sphere. The economy is seen as entirely constitutive of the nation, allowing little space for culture, society, or genuine political debate. Knowledges and capabilities that enable and enrich social, cultural, and political dialogues and actions are marginalised as not in the national interest through the price signalling. In the quest for technologically enabled economic growth, social sciences and humanities are relegated to the category of luxuries the nation cannot afford. The mission of building a ‘future-fit Australia’ is primarily left to the STEM fields. Progress requires much more than scientific knowledge and skills. As British philosopher Bertrand Russell (1950) said, ‘“Change” is scientific and “progress” is ethical’ (p.8). In contrast, the future imagined in the suite of JRG policies and press commentary is one in which a narrowly conceived, skills-based version of education (in which education is not distinguished from training) is instrumentally deployed in the services of the economy, specifically jobs.

The reform is also characterised by *issue omissions*. Within the context of *Industry 4.0* (Molla & Cuthbert, 2019), the political discourse has shifted from ‘knowledge economy and society’ to ‘digital economy and society’ (Australian Government, 2021). The future of work presupposes education and training that prepare individuals for lifelong learning and innovation. Industry 4.0 is future-ready in the sense that it draws on AI, cloud computing, and other emerging technologies, but it requires specific talent that combines judgment and agility (Accenture, 2021; Molla & Cuthbert, 2019). However, while the reform advocates for innovation, productivity, and competitiveness, issues of research and research training receive no attention. When the research capacity of universities is mentioned, the focus is on those institutions located in regional areas. Entrepreneurship and innovation rest on new knowledge created through basic research, produced mainly in universities and research institutions. Building an innovation-led economy without substantially supporting research and research training is unimaginable.

Furthermore, no reference is made to the perils of climate change, its impact on employment, public health, agriculture, water and energy, the environment, and all areas of social and economic activity. The reform fails to envision a critical role for the nation’s HE in protecting the environment (Barnacle & Cuthbert, 2021), providing the blueprint and the technologies for economic and industrial transformation that make possible a ‘green growth transition’ (OECD, 2017). One can only prepare graduates ‘for jobs of the future’ by ensuring that they learn how to be adaptable and agile lifelong learners. As an institution of knowledge production, ‘the good university’ (Connell, 2019) can play a vital role in re-imagining a post-carbon future (Bacevic, 2021). In this regard, the reform ignores the need for key Australian industries and the labour markets they support—primarily but not exclusively mining—to restructure to adapt to a rapidly decarbonising global economy. The role of the social sciences in understanding and enabling the cultural and social changes (in the labour market, in patterns of consumption) necessary for the Australian economy to decarbonise is omitted from this vision of the future.

The JRG reform rests on *a recycled imaginary* and a tired lexicon. The reform draws on a neoliberal policy logic (Ball, 2012; Rizvi, 2017; Rizvi & Lingard, 2011) that has dominated HE policy discourses in the last four decades but now shows signs of wearing thin even with its former proponents. The Government uses the pandemic as an opportunity to (re)articulate its pre-existing neoliberal policy agendas of *privatising* and *economising* HE. The reform cuts public spending on selected courses. For example, as the reform significantly reduced public funding per student, additional private contribution is expected to reach around $414 million per year (Littleton, 2022). The reform also calls on the university sector to support the nation’s economic goals of productivity and competitiveness by producing more graduates in selected fields. In other words, the Government used the pandemic-induced shock to introduce legislative measures that it failed to do so three times before the COVID-19 crisis. This crisis instrumentalism echoes O’Connor’s (1988) idea that ‘crisis brings into being new forms of flexible planning and planned flexibility’ (p.29). The crisis narrative that guided the JRG reform does not trigger new imaginaries.

A crisis is not just a moment of profound rupture that presents a challenge to the legitimacy of established policy directions; it also ‘creates pressure to devise new coping methods’ (Diamond, 2019, p.iii). A crisis demands new social imaginaries. Taylor (2004) identifies two pathways of imaginary formation: new theories that inspire new practices or reinterpretation of existing practices that lead to a new vision of the future. During a crisis, as our analysis shows, those in power may also choose to enact pre-existing imaginaries, responding to new challenges with old answers.

For many commentators (e.g. Azmanova 2020; Davidson, 2017; Haiven, 2014; Kaun, 2016; Klein, 2008), capitalism has a built-in tendency to crisis. Klein (2008) observes that crises (e.g. wars, coups, terrorist attacks, market crashes, or natural disasters) create an opportunity for neoliberal actors to impose unpopular policies when people are vulnerable and distracted. She uses the term ‘shock doctrine’ to describe ‘the quite brutal tactic of systematically using the public’s disorientation following a collective shock’ in order to ‘push through radical pro-corporate measures’ (p.56). Azmanova (2020) specifically argued that the combination of labour market liberalisation and the reduction of social spending makes the crisis a permanent feature of neoliberal capitalism. Also, notwithstanding the magnitude of the crisis, a total collapse of the system is improbable: ‘Government steps in when the going gets rough, ensuring that wealthy risk-takers will be bailed out in the worst of times’ (Hughes, 2021, para.4). But the COVID-19 pandemic appears to present a departure point. Many proponents of unfettered market rationality declared the fracture of the neoliberal order. The Financial Times Editorial (2020) declared that COVID-19 laid bare ‘the frailty of the social contract’ and called for governments to (a) commit to ‘radical reforms’ that reverse the neoliberal policy direction and (b) ‘accept a more active role in the economy’. Likewise, Schwab and Malleret (2020) stressed the importance of moving on from the free market fundamentalism (or neoliberalism) that bred much of our time’s socioeconomic and political ills. In this regard, the JRG reform is at odds with growing scepticism towards the neoliberal policy logic. In emphasising market-based *competition*, personal *choice*, and human *capital*, the reform overlooked the importance of civic *cooperation*, shared *commitment*, and human *capability*. The neoliberal policy reasoning pays little attention to education as a common good: if you fail to invest in your own education, you are likely to miss out economic opportunities, and that puts the blame on you.

Elements of the reform are also *nativist in orientation*. This nativism echoes the populist anti-globalisation sentiment which is on the rise globally and was articulated by Australian Prime Minister Scott Morrison in his resistance to ‘negative globalism’ (Murphy & Doherty, 2019). The nativist vision seeks to return to a period in which the HE system was not dependent on the revenue generated by international students’ fees without providing the funding which universities turned to international tuition fees to replace. Universities are imagined only as places of teaching and the students as exclusively Australian. Elements of this imaginary evoke an earlier exclusionary, racist, and xenophobic period of Australian history, including the White Australia regime that persisted for much of the twentieth century (from the 1900 to 1970s). The reform reflects the anti-immigration agenda of the ruling Coalition and the views of populist politicians such as Pauline Hanson’s One Nation Party, who have been pointedly critical of the numbers of international students at Australian universities.

The nativist orientation contradicts the recovery agenda. On the one hand, the Government underscores skills urgency and the implications for economic productivity and competitiveness. On the other, the call is for home-grown STEM graduates; the future depends on ‘…on knowledge-intensive jobs *held by Australians*’ (DESE, 2020a, p.3). The push for an HE sector that serves the nation, domestic students, and a vision of a technologically enabled economy seeks to reverse Australia’s historical dependence on immigration to compensate for a skills deficit, especially in STEM fields and especially for the mining sector. In reality, international students represent the nation’s largest source of skilled migrants (Horne, 2020).

When it comes to the reform’s equity agenda, there is an overlap between nativist approaches and reductionist accounts. The complex issue of equity is reduced to a simple urban/rural disparity. From the 1990s to the early 2010s, the Government identified six equity groups that were ‘significantly under-represented’ in Australian higher education: Aboriginal and Torres Strait Islander people, women (in non-traditional courses and postgraduate study), people from low socioeconomic backgrounds, people with disabilities, people from non-English-speaking backgrounds (NESB), and people from rural and isolated areas. In 2012, the Government narrowed down the number of equity groups from six to three: ‘those students who are Indigenous, who come from a low-SES background, or who have a disability’ (Australian Government, 2012, Sect. 1.5.1). In the JRG reform, the equity provision is characterised by regionalism. Intersectional indicators of educational disadvantage are reduced to the very comfortable Australian understanding of the city versus the bush. Rurality is commonly associated with nativist images that embody the cultural values of White Australians.

Sargeant (2021) speaks of crisis as ‘a moment of discontinuity’ that requires ‘an act of imagination large enough to *envisage a future* different from the continuities mandated by the past, and powerful enough to *generate a strategy* sufficient to chart a path towards this future’ (p.5, emphasis added). During the consultation period, some commentators were optimistic about the transformative role of the reform. In his commentary on the Bill, Buchanan (2020) noted, ‘This is *the end point of the small government* […] the 30-year experiments with markets and privatisation in post-school education have failed’ (emphasis added). However, as is evidenced in the actual reform, such optimism appears to be unwarranted. The reform does not usher in a *policy paradigm shift* (Hall, 1993) as it continues to champion the pre-pandemic agendas of economising HE and cutting public spending. It puts forward neither a novel imagining of the future nor a novel policy agenda. It is trapped in the cycle of providing a single solution to complex (and ill-defined) problems. In addition to being exclusive, this singular view is also politically dangerous. As Eisner (2017) posits, ‘the denial of complexity is the beginning of tyranny’ (p.147). A democratic social order rests on diverse imaginaries and inclusive political processes.

## Conclusions

We set out to understand how the Australian Government employed a crisis discourse to introduce major HE legislative reform and articulate futures that the reform aims to realise. The analysis identifies four policy problem areas: skills shortage, weak industry linkage, population growth, and geographic disparities in higher education participation. In response, the Government outlines specific strategies, including price signalling, industry collaboration, expansion of university places, and regional funding. Students electing non-preferred fields will pay punitive fees for their study choices. The imagined futures that those policy measures seek to achieve may be framed as an *economically competitive nation* and *a socially just higher education sector*. However, a closer examination of the reform reveals a recycled policy imaginary, reductionist tendencies, nativist orientation, and the omission of significant issues (arguably constitutive of crises themselves).

In the last four decades, the machinery of education policymaking has been lubricated by the economic logic of competitiveness, productivity, and innovation. In this regard, nothing new has been envisioned in this reform—the focus is still on narrow *human capital* accounts of HE. Instead of re-envisioning a new future, we argue, the JRG reform reactivates the ‘neoliberal imaginary’ that ignores the value of education as a common good. The reform follows the usual (and often superficial) act of balancing *economic goals* (producing job-ready graduates for competitiveness) and *social demands* (equity measures that target regional and Indigenous students). In essence, the JRG reform signifies what Boin et al. (2008) refer to as ‘crisis exploitation’. The Government framed the COVID-19 pandemic and the associated fear of an economic downturn as a *crisis* to sell old policy packages and imaginaries.

Reforms anchored to old and tired imaginaries are unlikely to be transformative. As Chen Qiufan puts it: ‘with every future we wish to create, we must first learn to imagine it’ (Lee & Qiufan, 2021, p.xxi). The issue is not with expecting universities to be responsive and adaptive to the nation’s needs and priorities. Instead, an excessive emphasis on technical training risks the emergence of generations with little or no regard for democracy, social cohesion, and environmental justice. Technological changes indeed raise the skill requirements of jobs, but human capital (the educational attainment of the workforce) is a narrow parameter of progress. A cohesive, prosperous, and free society is much greater than the economic activities underpinning it.

## Footnotes

1 Although the beginning of the neoliberal crisis can be traced back to the 2008 financial recession, the COVID-19 pandemic appears to be a ‘tipping point’. Now ‘big government’ is not a big issue for those on the Political Right. On 4 April 2020, the British-based business daily *Financial Times*, a leading proponent of an unfettered market ideology, published an editorial that advocated abandoning neoliberalism as an ideology. The editorial partly reads: ‘Radical reforms – reversing the prevailing policy direction of the last four decades – will need to be put on the table. Governments will have to accept a more active role in the economy. They must see public services as investments rather than liabilities and look for ways to make labour markets less insecure’ (para #6). Likewise, writing for *Time* magazine (26 April 2021), economist and co-founder of Facebook Chris Hughes declared ‘the free market is dead’. If a crisis in confidence in government triggered the reign of the free market fundamentalism, he wrote, a crisis in the unfettered market has increased trust in government.

2 See https://www.afr.com/politics/peter-costellos-baby-bonus-generation-grows-up-20170831-gy7wfg.
